# Balancing Selection for Pathogen Resistance Reveals an Intercontinental Signature of Red Queen Coevolution

**DOI:** 10.1093/molbev/msab217

**Published:** 2021-07-21

**Authors:** Yann Bourgeois, Peter D Fields, Gilberto Bento, Dieter Ebert

**Affiliations:** Zoology, Department of Environmental Sciences, University of Basel, Basel, Switzerland

**Keywords:** *Daphnia magna*, coevolution, *Pasteuria ramosa*, negative frequency-dependent selection, Red Queen, population genomics

## Abstract

The link between long-term host–parasite coevolution and genetic diversity is key to understanding genetic epidemiology and the evolution of resistance. The model of Red Queen host–parasite coevolution posits that high genetic diversity is maintained when rare host resistance variants have a selective advantage, which is believed to be the mechanistic basis for the extraordinarily high levels of diversity at disease-related genes such as the major histocompatibility complex in jawed vertebrates and R-genes in plants. The parasites that drive long-term coevolution are, however, often elusive. Here we present evidence for long-term balancing selection at the phenotypic (variation in resistance) and genomic (resistance locus) level in a particular host–parasite system: the planktonic crustacean *Daphnia magna* and the bacterium *Pasteuria ramosa*. The host shows widespread polymorphisms for pathogen resistance regardless of geographic distance, even though there is a clear genome-wide pattern of isolation by distance at other sites. In the genomic region of a previously identified resistance supergene, we observed consistent molecular signals of balancing selection, including higher genetic diversity, older coalescence times, and lower differentiation between populations, which set this region apart from the rest of the genome. We propose that specific long-term coevolution by negative-frequency-dependent selection drives this elevated diversity at the host's resistance loci on an intercontinental scale and provide an example of a direct link between the host’s resistance to a virulent pathogen and the large-scale diversity of its underlying genes.

## Introduction

Hosts and parasites engage in specific interactions that are believed to select for and maintain genetic diversity at host resistance genes (Sackton et al. 2007; Ebert and Fields 2020; Radwan et al. 2020). If pathogens evolve to overcome the resistance of common host alleles, rare resistance alleles have a selective advantage until they also become common. This form of time-lagged negative-frequency-dependent selection (NFDS), often referred to as Red Queen coevolution, is believed to increase genetic polymorphism at loci that interact with the antagonist (Charlesworth 2006; Thrall et al. 2015; Rabajante et al. 2016). Indeed, the Red Queen hypothesis has gained so much popular support that regions in host genomes that show elevated genetic diversity are taken as potential indicators of antagonistic coevolution, even when the coevolving antagonists are unknown. The Red Queen model was originally conceived to be a process that acts within populations, but host–parasite interactions undergoing NFDS also shape genetic diversity among populations (reviewed in Ebert and Fields [2020]). Because resistance alleles that migrate into host populations are rare, they may be favored by selection, resulting in a higher effective migration rate than other alleles in the genome (Charlesworth et al. 1997; Thrall et al. 2012; Jousimo et al. 2014; Bolnick and Stutz 2017). Nevertheless, the random loss of genotypes in small populations and strong selection from local parasites can also quickly lead to genetic divergences between neighboring populations (Lively and Dybdahl 2000; Bourgeois et al. 2017). Given this combination of regional and local dynamics, even nearby populations can display high divergence at resistance loci, whereas distant populations may show low divergence (Charlesworth et al. 1997). On large geographic scales, thus, one would expect genomic regions with resistance loci involved in coevolution to display signatures of higher genetic diversity than the rest of the genome, balancing selection, and reduced spatial structure. Evidence for these predictions has been found in the vertebrate MHC loci (Eizaguirre et al. 2012; Kaufman 2018) and in R-genes in plants (Bergelson et al. 2001; Tellier and Brown 2011), although, for both these groups of genes, the functional link between the resistance genes and the long-term coevolving parasites is missing. In other systems, although coevolutionary dynamics between hosts and specific parasites have been demonstrated, the underlying genetics are not known (Thrall et al. 2012; Gibson et al. 2018).

**Fig. 1.**
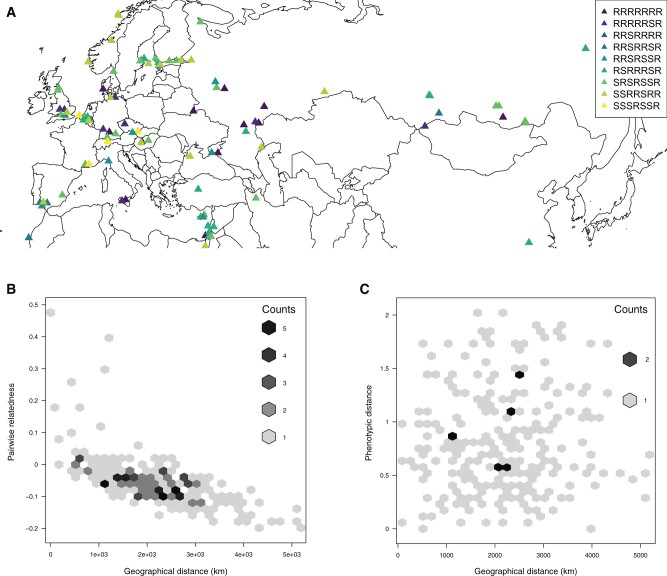
(*A*) Resistotypes designations for the 125 *Daphnia magna* clones from across Eurasia and North Africa used in this study. Seven-letter codes indicate R (resistant to spore attachment) or S (susceptible) for the following parasite clones (in order): C1, C19, P15 (hindgut attachment), P15 (foregut attachment), P20, P21 (hindgut attachment), and P21 (foregut attachment). To improve readability, only resistotypes found at least four times are shown. (*B*) Plot of relatedness using genomic SNP data for 23 clones sampled from the same populations as in B against their pairwise geographic distance. Counts indicate overlaying data points. (*C*) Plot of pairwise geographic distance and pairwise distance of resistance phenotypes for 23 *D. magna* populations. Phenotypic distance is measured as the pairwise Euclidean distance incorporating population differences in the frequencies of resistotypes.

Here we test the hypothesis that host–parasite coevolution causes balancing selection at a host resistance gene cluster in the water flea *Daphnia magna*, coevolving with the obligate bacterial endoparasite *Pasteuria ramosa*. In this system, both the host and the parasite have a wide natural distribution covering nearly the entire Holarctic. Infections bear extreme fitness costs for the host (Luijckx et al. 2012). Resistance follows a matching allele model, preventing individual hosts and parasite genotypes from reaching fixation (Luijckx et al. 2013), and displays high diversity within populations (Andras and Ebert 2013). Coevolution has been indicated in this system based on a study of sediment cores showing the temporal dynamics of *D. magna*–*P. ramosa* interactions over about three decades (Decaestecker et al. 2007). To test for predicted patterns of genetic diversity within and between populations, we used a panel of *D. magna* genotypes consisting of single clonal lines collected from 125 populations in Eurasia and North Africa (fig. 1*A*), each with information about geographic origin and genome sequences. Notably, for each host genotype we also possessed resistance phenotype data for five parasite genotypes. To test for signatures of balancing selection, we analyzed patterns of diversity at both the phenotypic and genetic level. The latter focused especially on a genomic region in *D. magna* that explains the most variance in its resistance to *Pasteuria* (Bento et al. 2017). This region (positions 1,368,860 to 1,506,215 on scaffold00944 of the *D. magna* reference genome, version 2.4, here called “resistance QTL”) contains a supergene that has been found to harbor extremely diverged haplotypes (Bento et al. 2017). Evidence from several sources suggests that this region plays a role in resistance to *P. ramosa* in natural populations. Genome scans for selection and association show a significant signal for this cluster and its flanking genomic regions across European *D. magna* populations (Bourgeois et al. 2017). The same association signal is found within a single panmictic population in Switzerland (Ameline et al. 2021). It has historically been difficult to establish a functional link between resistance, genetic diversity, and the consequences for coevolution, as the underlying genes of either the coevolving parasites or the host were unknown. Nevertheless, the architecture of resistance to *P. ramosa* has now been characterized for *D. magna* (Routtu and Ebert 2015; Bento et al. 2017; Bourgeois et al. 2017; Ameline et al. 2021), making it possible to test directly for a signature of balancing selection at this region and understand how coevolution with a virulent and widespread parasite affects host genetic variability.

## Results and Discussion

### No Geographical Structure for Resistance Phenotypes

Parasite-driven NFDS is expected to result in a geographic mosaic of resistance phenotypes and genotypes across the host’s range with no or weak geographic structure as compared with the genetic background (Kaltz and Shykoff 1998; Tellier and Brown 2011; Ebert and Fields 2020). To test this, we investigated whether polymorphism for resistance phenotypes displayed a signal of spatial structure. We isolated five parasite strains (*P. ramosa* C1, C19, P15, P20, and P21) from natural populations across Europe (Luijckx et al. 2011) and phenotyped *D. magna* clones for resistance to these by assessing whether labeled spores attached to the host's foregut (all five parasites) or hindgut (two parasites: P15 and P21) (Duneau et al. 2011) (supplementary table S1, Supplementary Material online). This resulted in seven different resistance phenotypes, which we summarized with 7-letter codes (R for resistance and S for susceptibility for each of the five foregut and two hindgut phenotypes; fig. 1*A*). Note that this phenotypic assessment covers only a fraction of the total phenotypic variation and should be seen as a sample for the actual diversity in parasites and host resistotypes. Resistance phenotypes were found to be uniformly distributed across the entire study region without a pattern of isolation by distance (IBD; fig. 1*A* and supplementary table S2, Supplementary Material online). This was further confirmed by a global Distance-based Moran's eigenvector maps (dbMEM) analysis, which did not detect any significant positive spatial correlation in the spatial repartition of the seven resistotypes (_adj_*R*^2^ = 0.006, *P *=* *0.068). The same observation held when considering each resistotype independently (_adj_*R*^2^ between −0.12 and 0.002, all *P *>* *0.1). To understand the biogeographic context for this absence of a spatial pattern, we compared this analysis to a similar analysis using single-nucleotide polymorphism (SNP) data derived from the genomic sequences of the 125 *D. magna* clones. We found a strong pattern of IBD for genomic data, where average relatedness between individual host clones decreased with geographic distance (*N *=* *125, Mantel *R* = −0.56, 1,000,000 permutations, *P *<* *10^−6^). This pattern is consistent with a previous study of the same *Daphnia* species (Fields et al. 2015). Moreover, in *D. magna*, other phenotypic traits show a clear geographic structure (Yampolsky et al. 2014; Seefeldt and Ebert 2019), underscoring that the lack of geographic structure for *Pasteuria* resistance is unique.

We further examined whether resistance polymorphism (only to *Pasteuria* C1, C19, P15, and P20). The P21 isolate was isolated only later also held true on the single-population scale. To do so, we obtained resistance phenotypes for *D. magna* individuals hatched from resting eggs from 23 populations from the Western Palaearctic for which we could successfully phenotype at least five host genotypes—20 of them polymorphic (R or S) for at least one *Pasteuria* strain. There was no correlation between variation in resistotype frequencies and pairwise distance (fig. 1*C*) based on Mantel tests (supplementary table S3, Supplementary Material online). In contrast, SNP data across the genomes revealed strong IBD for the same 23 populations (fig. 1*B* and supplementary table S3, Supplementary Material online). This lack of positive spatial correlation was confirmed by a dbMEM analysis (_adj_*R*^2^ < 0). Thus, phenotypic diversity for resistance against *Pasteuria* infections, which did not show a spatial pattern, contrasted strongly with the genomic background, which was shaped by IBD. This uniform diversity in resistance on a very large geographic scale (entire Palaearctic) coincides with the theory of host–parasite coevolution by balancing selection, which projects that phenotypic diversity is maintained at loci of functional importance for the interaction of the antagonists. To our knowledge, this finding has not been shown before for phenotypic traits under coevolution.

### Genomic Data Reveal a Signature of Population Structure and Postglacial Expansion

**Fig. 2.**
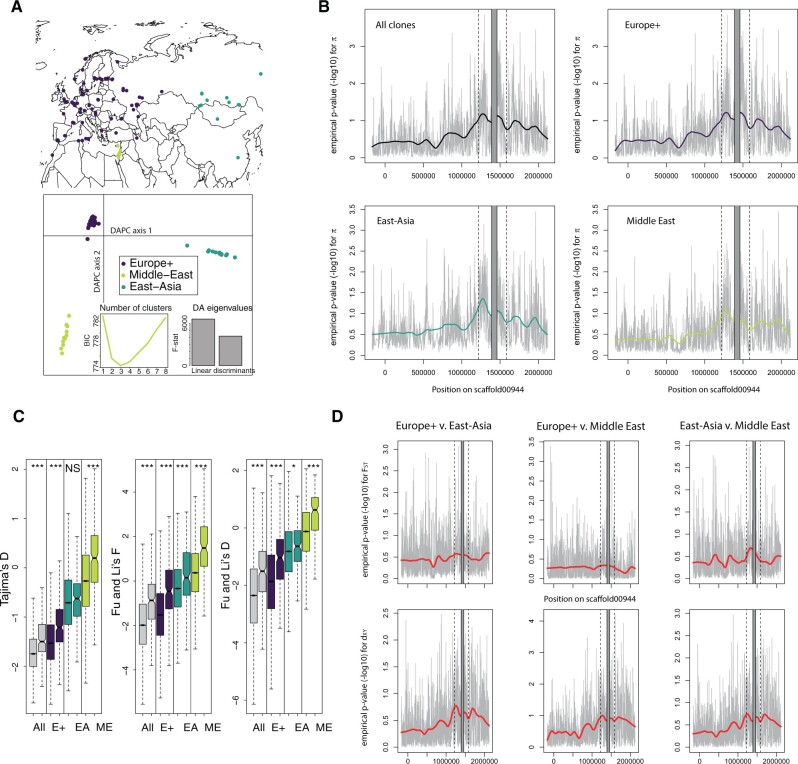
Genetic diversity and population genetic parameters in the genomic region flanking the *D. magna’*s resistance QTL. (*A*) Sites of origin and DAPC on 8,978 genome-wide SNPs with no missing data sampled every kb for 125 *D. magna* genotypes. The DAPC analysis identified three major groups: Europe+ (E+), East-Asia (EA), and Middle-East (ME). (*B*) Empirical *P* values for nucleotide diversity in 1-kb windows for all 125 *D. magna* clones and the three geographic groups. Diversity statistics are ranked in decreasing order to obtain *P* values, so low *P* values correspond to high diversity. The resistance supergene region (QTL locus ± 100 kb) is located between the two dotted lines. The supergene itself is masked in gray due to very poor mapping of short reads to this region (positions 1,435,000 to 1,490,000 on scaffold00944). Coordinates correspond to *D. magna* 2.4 genome. Negative coordinates correspond to a region in the PacBio scaffold that mapped outside the original scaffold00944 (see supplementary fig. S1, Supplementary Material online). (*C*) Neutrality statistics (over 1-kb windows) in the region around the resistance supergene compared with genome-wide values (excluding scaffolds shorter than 10 kb in genome version 2.4). In all pairwise comparisons, the boxplots on the left and right correspond to the genomic background and the region around the resistance supergene, respectively. For Fu and Li’s *F* and Fu and Li’s *D*, *Daphnia similis* was used as an outgroup; higher values are associated with frequency spectra skewed toward ancestral variants and alleles at intermediate frequencies, supporting balancing selection. *P* values were obtained from Wilcoxon rank-sum tests (NS: nonsignificant; *: *P *<* *0.05; ***: *P *<* *0.001). Color codes as in figure 2. (*D*) Empirical *P* values for divergence statistics. The upper panels show the *F_ST_*, which is expected to be reduced if balancing selection is present, for all three pairwise comparisons among the geographic regions Europe+, East-Asia, and Middle-East. In that case, *F_ST_* values are ranked in increasing order to obtain the empirical *P* value. The lower panel shows the absolute divergence, *d_xy_*, for the same pairs, which is expected to increase if there are ancient polymorphic alleles.

To investigate patterns of genetic diversity and divergence, population structure and history should be taken into account. Using a discriminant analysis on principal components (DAPC; Jombart et al. 2010) based on genotypes (supplementary fig. S1*A*, Supplementary Material online) for all 125 *D. magna* clones, we identified three geographic clusters. As the model with three clusters had the lowest Bayesian Information criterion (BIC), we assigned individuals to three putative geographical clusters, which we called, for simplicity, Europe+, Middle East, and East-Asian (*N *=* *100, 11, and 14, respectively). Our results supported previous studies in revealing substantial divergence between East-Asian samples and the other groups (Fields et al. 2015, 2018); indeed the East-Asian cluster was clearly separated from other clones by the first discriminant function with the highest eigenvalue (fig. 2*A*). In addition, estimates of differentiation measured by *F_ST_* over 1-kb windows along the genome (see Materials and Methods) were also substantially higher for the East-Asian cluster (average *F_ST_* = 0.32, 0.37, and 0.124 for Europe+ vs. East-Asia, Middle-East vs. East-Asia, and Europe+ vs. Middle-East, respectively, Wilcoxon signed rank tests, all *P *<* *2.2 × 10^−16^).

Past reductions in effective population size can produce genome-wide signatures that are similar to balancing selection (Charlesworth 2006). Indeed, demographic analyses reveal a clear signature of expansion and population splits following the last glacial maximum (supplementary fig. S1 and table S4, Supplementary Material online for exact point estimates and confidence intervals), which coincides with previous studies based on mitochondrial data (Fields et al. 2018). Such demographic events are thought to skew the genome-wide allele frequency spectrum (AFS) toward more rare alleles, whereas balancing selection would maintain alleles at higher frequency. The absence of a strong recent bottleneck suggests that false positive evidence for balancing selection due to demography most likely do not explain the patterns we observed near the resistance QTL.

### High Nucleotide Diversity and Skewed Allele Frequency Spectra near the Resistance Locus

To first assess whether the region around the resistance QTL displayed elevated nucleotide diversity, as would be expected under balancing selection (Charlesworth 2006), we improved the quality of scaffold00944 by using a PacBio contig from the same individual used to build the reference genome (supplementary fig. S2, Supplementary Material online), as described in a previous study (Bento et al. 2017). Nevertheless, divergence between variants within the supergene (roughly located between positions 1,435,000 and 1,490,000 on scaffold00944) were so high that alignment of short Illumina reads on the reference was not possible. Because the supergene haplotypes are not homologous and are difficult to assemble due to their repeat richness, typical population genetic approaches that rely on the alignment of diverged haplotypes are impossible for this region (Bento et al. 2017). In all subsequent analyses, thus, we excluded the supergene region to avoid the unreliable mapping of reads and instead focused on its flanking regions. As a result, our divergence measures and other population genetic summary statistics based on SNP variation in these flanking regions strongly underestimate the actual polymorphism in the region of highest diversity. All geographic clusters displayed a considerable increase in nucleotide diversity for 1-kb windows between positions encompassing the resistance QTL and the following 1 Mb (fig. 2*B*), with most windows in the top 5–10% genome-wide, and peaks in the top 0.1%. For all clones as well as for the three geographic clusters, nucleotide diversity was higher at the resistance region (hereafter defined as the resistance QTL ±100 kb), than the rest of the genome (Wilcoxon rank-sum tests, all *P *<* *2.2 × 10^−16^).

We then tested the hypothesis that alleles with intermediate frequencies should be more common in genomic regions under balancing selection than in the genomic background (Charlesworth 2006). Indeed, the resistance region showed an abundance of alleles at intermediate frequencies with a significantly elevated Tajima’s *D* (fig. 2). Using the closely related species *Daphnia similis* (Cornetti et al. 2019) as an outgroup to define ancestral alleles, we further found elevated Fu and Li’s *F and D* (Fu and Li 1993), indicating an excess of ancestral polymorphisms at intermediate frequencies (fig. 2*C*). This supports the hypothesis that balancing selection acts at the resistance region. We also note that genome-wide values for Tajima’s *D* were generally negative for the Europe+ and East-Asia clusters, and closer to 0 for the Middle-East cluster, consistent with the recent expansion inferred by our demographic analyses and evidence on mitochondrial genomes (Fields et al. 2018). The glacial refugium for the European *D. magna* was suggested to be in South-Eastern Europe/Middle East (Fields et al. 2018).

### Low Relative but High Absolute Measures of Spatial Differentiation

Another hallmark of balancing selection is reduced differentiation at selected loci among populations at large spatial scales. Migrating alleles at loci under balancing selection are likely to be rare upon arrival, giving them an advantage and, as a consequence, increases their effective migration rate. Neutral alleles, on the other hand, would only increase in the recipient population if they hitchhike with alleles under selection (Laine et al. 2011; Thrall et al. 2012; Phillips et al. 2018; Ebert and Fields 2020). In addition, alleles under balancing selection are less likely to go extinct in a given population because they are advantageous when rare. Balancing selection, thus, can be expected to reduce the turnover rate of alleles and to facilitate long-term persistence of polymorphism within populations (Charlesworth 2006; Leffler et al. 2013). Distinct populations would share polymorphisms and show reduced estimates of population differentiation at the genes under selection (Charlesworth 2006; Rico et al. 2015). To test this theory, we estimated genome-wide variation in relative (*F_ST_*) and absolute (*d_XY_*) differentiation using the three geographic clusters determined by the DAPC (fig. 2*A*). Introgression and selection are both expected to impact population differentiation and estimates of *F_ST_ and d_XY_* in different ways: balancing selection should decrease *F_ST_* and increase *d_XY_*, whereas recent introgression would reduce both statistics, as it is an absolute measure of divergence that captures the number of sequence differences since the most recent common ancestor (TMRCA) (Cruickshank and Hahn 2014). Our data clearly support balancing selection, showing reduced relative population divergence (significantly lower *F_ST_* for Europe+ vs. East-Asia and East-Asia vs. Middle-East comparisons; Wilcoxon rank-sum test, all *P *<* *2.2 × 10^−16^, fig. 2*D*), and increased absolute population divergence in the resistance region (higher *d_XY_* compared with genome-wide mean [all *P *<* *2.2 × 10^−16^, fig. 2*D*]). This suggests that alleles in the resistance region have a higher chance of spreading across populations and being maintained within populations, consistent with an advantage for being rare.

### Observed Patterns of Diversity Are Consistent with Simulations under Negative-Frequency-Dependent Selection but Not with Neutrality

**Fig. 3.**
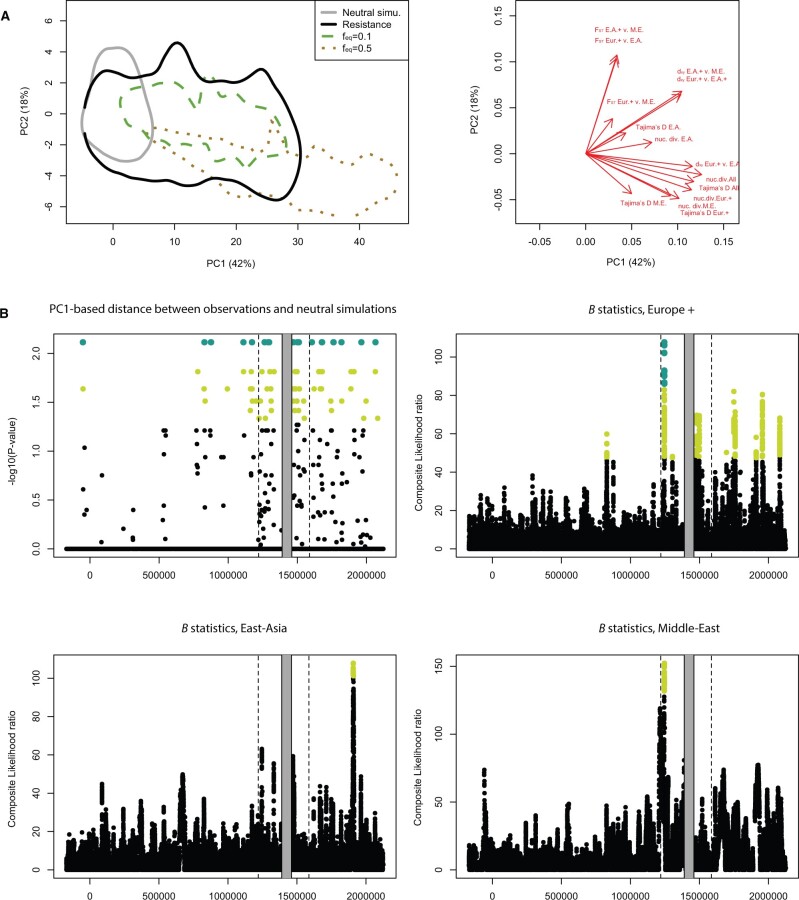
Comparisons of diversity between the resistance region, simulations, and the rest of the genome. (*A*) Principal components analysis (PCA) of 10 million neutral coalescent simulations. The statistics used include nucleotide diversity, pairwise divergence statistics, and Tajima’s *D* (correlation circle displayed in the right panel). Predicted values for the resistance region and two sets of SLiM3 NFDS simulations are also shown. The SLiM3 simulations were obtained with a fraction of new mutations recruited by selection of 0.1% and equilibrium frequencies (*f_eq_*) of 10% and 50%. The envelopes cover 95% of points from each category. (*B*) The upper-left panel shows Bonferroni-corrected *P* values obtained from comparing observations and neutral coalescent simulations for each 1-kb window. Light green points indicate *P* values < 0.05 and large dark green dots indicate *P* values < 0.01. The three other panels show the *B_0,MAF_* statistics. Composite likelihood ratio for each of the three geographic groups. The statistics compares local allele frequency spectra to the genome-wide spectrum and compares the likelihood of a model with balancing selection against a neutral model. Light green points indicate the highest 1% of scores genome wide, whereas large dark green dots indicate those among the top 0.5%.

To test whether differentiation and diversity statistics deviated significantly from neutral expectations, we generated 10 million coalescent simulations under our demographic model. We then performed a principal component analysis (PCA) on summary statistics (fig. 3*A*). The first PC axis (PC1) explains 42% of the total variance, and is clearly correlated with diversity statistics and Tajima’s *D*. The second axis (PC2) explains 18% of the variance and is mostly correlated with *F_ST_*. Predicted values for the resistance region are consistent with our previous observations, with high PC1 scores (high diversity and Tajima’s *D*), and low PC2 scores (low *F_ST_*). There is a high density of windows deviating from neutrality in the resistance region (fig. 3*B*). This is further confirmed by a scan for balancing selection using the *B_0,_*_*MAF*_ statistics (Cheng and Degiorgio 2020). The test contrasts allele frequency spectra around a focal SNP to the genome-wide frequency spectrum to estimate the likelihoods of models with and without balancing selection, under a broad range of equilibrium frequencies. We observe clear signals in Europe+ and Middle-East clusters (fig. 3*B*), with many regions above the top 1% genome-wide threshold.

To test the conditions under which NFDS could produce the observed patterns of diversity, we ran simulations using the forward-in-time simulator SliM3 on the demographic history estimated from whole-genome data. We simulated 1-kb windows with mutation and recombination rates consistent with current knowledge about *D. magna*. We varied the fraction of new mutations recruited by selection and the equilibrium frequency at which balanced polymorphisms are maintained. As expected in NFDS simulations, nucleotide diversity, Tajima’s *D*, and *d_XY_* were higher than in neutral simulations, whereas *F_ST_* was lower (fig. 3*A* and supplementary fig. S3, Supplementary Material online). In scenarios where 0.01% of new mutations were recruited by selection, diversity was generally lower than our observations in the resistance region. However, the skew in the frequency spectrum (Tajima’s *D*) was consistent with observations. A closer match between simulations and observed data occurred in scenarios where 0.1% of new mutations were recruited by selection with an equilibrium frequency of 0.1 (fig. 3*A*). For higher equilibrium frequencies, Tajima’s *D* values were much higher than our observations (supplementary fig. S3, Supplementary Material online), consistent with a stronger skew of the AFS toward higher frequencies. This suggests that balanced polymorphisms in the resistance region may not necessarily reach very high frequencies at the geographical scale considered here, which is consistent with a fast tracking of host genotypes by quickly evolving pathogens that rapidly reduce the selective advantage of the most common resistotypes (Decaestecker et al. 2007).

It is important to note that we scaled down effective population sizes and times by 100 in our simulations to ensure fast running times, which limits the maximum strength of selection that we could simulate. We assumed a selective coefficient of 0.005 for newly established mutations under NFDS before scaling parameters. In actual populations, the strength of selection is likely higher, as *P. ramosa* castrates the host, reducing the residual fitness of the infected female by about 90% (Ebert et al. 2016). Such strong selection should counteract the effects of recombination over large genomic intervals, especially given the large effective population sizes considered here. In that case, an even lower fraction of mutations recruited by selection would be enough to generate the patterns of diversity observed in the resistance region. Although it seems clear that neutrality can be rejected, further simulations at multiple spatial scales would be needed to properly compare various NFDS scenarios. Nevertheless, our simulations suggest that even moderate selection and low equilibrium frequencies can lead to a marked increase in diversity in our system.

### The Resistance Region Displays an Excess of Ancient Alleles and Older Coalescence Times

**Fig. 4.**
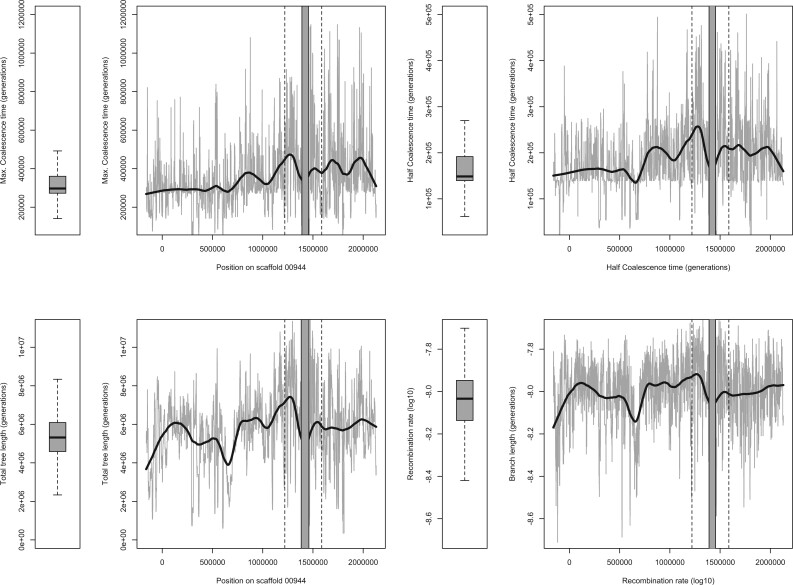
Coalescence analysis for 1-kb windows across the resistance region (indicated by flanking vertical dotted lines). Coalescence times are given in equivalent generations (sexual + asexual). Approximate times in years can be obtained by dividing by ten, assuming ten generations a year. Boxplots summarize the distribution of statistics from two other large scaffolds (00024 and 00512) totaling more than 6 Mb. Half-coalescence time is defined as the minimum time at which half of the lineages coalesce (see main text). Total tree length corresponds to the average sum of all branches in genealogies of nonrecombining blocks. Recombination rates are estimated by ARGWeaver and log10 transformed.

Reduced allele turnover during balancing selection implies that alleles have a longer lifespan. Thus, regions under long-term balancing selection should display older coalescence times and higher local effective population sizes (Charlesworth 2006). Our findings of higher absolute divergence (*d_XY_*_,_fig. 2*C*) in the resistance region suggest that the alleles in this region are already older than alleles in other parts of the genome. We confirmed this by computing ancestral recombination graphs (ARG) (Rasmussen et al. 2014) on a set of three large scaffolds, including the scaffold with the resistance region, retrieving local genealogies at all nonrecombinant blocks. We restricted the analysis to 48 host clones that had fewer than 5% missing genotypes to achieve a high quality of the estimates and to reduce computational burden (we could not handle with the entire genomes of all genotypes). We also included priors on past changes in effective population sizes based on our demographic analyses. The region displaying a local reduction in *F_ST_* also displayed longer times to the TMRCA (coalescence time; fig. 4), further supporting long-term balancing selection at this region. This long coalescence time was not driven by a few older haplotypes, since the half coalescence time (HCT; the minimum time at which half of lineages coalesce) was also substantially older than the background (fig. 4).

Higher diversity near the resistance region may be caused by the reduced influence of linked selection if recombination rates are particularly high (Charlesworth 2013; Cruickshank and Hahn 2014; Burri 2017). While in the upper range when compared with other scaffolds, recombination rates estimated by ARGWeaver in the resistance region are not extreme (fig. 4 and supplementary fig. S4, Supplementary Material online), suggesting that higher recombination rates and weaker linked selection do not explain the locally high concentration of windows of high diversity.

### Genome Scans for Ancient Balancing Selection Pinpoint Genes Involved in Glycolipid and Glycoprotein Synthesis

**Fig. 5.**
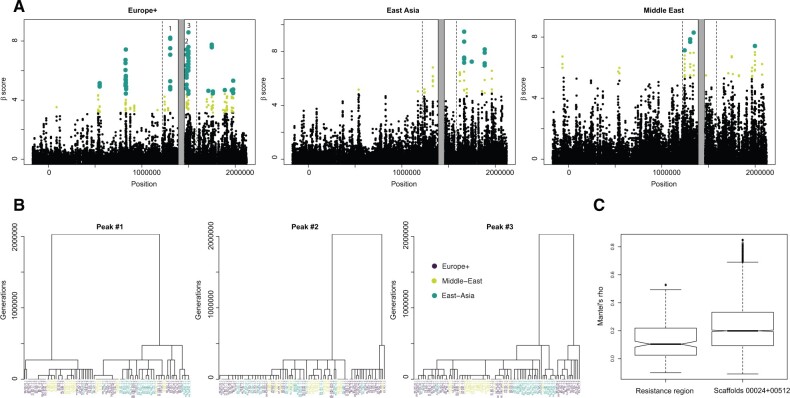
Scan for balancing selection in the resistance region and flanking sites. (*A*) Results from the Beta scan analysis. Light green points indicate the highest 1% of scores genome wide, whereas large dark green dots indicate those among the top 0.5%. (*B*) Local topologies obtained from ARGWeaver for nonrecombining blocks overlapping with SNPs at the three peaks are highlighted in (*A*). (*C*) Mantel’s correlation coefficients obtained by comparing the matrix of geographical distance between clones with 5,000 matrices of phylogenetic distance inferred from 5,000 trees randomly sampled across scaffolds of the genome.

It is predicted that the flanking regions surrounding a genomic region under balancing selection will show little signature of balancing selection because older polymorphisms give recombination enough time to erode a signal on the flanking regions (Charlesworth 2006). However, our study found clear evidence of signals of balancing selection in the flanking region of the resistance supergene, hinting that further polymorphisms in these flanking regions might be under balancing selection. Furthermore, an earlier resistance mapping study also suggested that genes outside the resistance supergene are linked to phenotypic variation in resistance (Bourgeois et al. 2017). We therefore conducted genome scans of balancing selection using recently developed *β* statistics (Siewert and Voight 2017) to investigate at a higher resolution which regions near the resistance supergene displayed the strongest signals of selection. *β* should be sensitive to alleles at an equilibrium allele frequency between 0.2 and 0.5, covering the range of equilibrium frequencies explored in our simulations. It should also be robust to the lower sample sizes in the Middle-East and East-Asia clusters (Siewert and Voight 2017). We also expect *β* to be more powerful than other statistics such as *NCD* (Bitarello et al. 2018), which mostly target alleles with equilibrium frequencies in the 0.3–0.5 range. We found several signals of balancing selection in the region around the resistance supergene, particularly in the Europe+ lineage where our sample size was largest (fig. 5*A*). We then extracted genealogies from the ARGWeaver output for nonrecombining blocks that overlapped the three main peaks identified in the targeted region (fig. 5*B*). These genealogies displayed very long branches with very old coalescence times (∼2 million generations). For two (#1, #2) of the three peaks of beta score close to the resistance QTL, clones from all three geographic regions were represented in both clades. For the third peak (#3), relatedness between non-European clones coincided with the clusters identified by our DAPC analysis, with individuals from the same genetic group clustering together in the phylogeny. This lack of strong geographic structure was common along the resistance region. Correlations between geographic and phylogenetic distance between clones were weaker for hundreds of ARGWeaver trees randomly sampled in the resistance region than for trees sampled in other scaffolds (fig. 5*C*, Wilcoxon rank-sum test, *P *<* *4.3 × 10^−14^), consistent with the maintenance of ancestral polymorphism in the resistance region.

We then identified which genes near the resistance region were associated with *β* scores higher than the top 1% genome-wide (table 1). These genes displayed homology with glucosyltransferases, chitinases, and transcription factors, echoing the function of genes previously found in the two haplotypes of the resistance supergene (Bento et al. 2017). Other peaks on the scaffold outside the resistance supergene itself were mostly concentrated between positions 1,820,000 and 2,000,000 on scaffold00944 and overlapped genes with similar annotations (table 1), suggesting that the resistance QTL may belong to a larger region recruited by NFDS. We also found genes annotated as digestive enzymes such as trypsins and serine-proteases, an interesting observation given that *P. ramosa* starts its infection process in the host's oesophagus and hindgut (Duneau et al. 2011; Bento et al. 2020) and attaches to host cells through collagen-like proteins (Mouton et al. 2009; Andras et al. 2020).

**Table 1.** List of candidate genes with a signature of balancing selection on scaffold00944, highlighting the geographical clusters in which they were identified.

Start	End	Gene Name	Populations with Outlier *β* Score	Region	ME Max LR	EA Max LR	E+ Max LR
166339	169222	Noncoding RNA	E+	scaffold00944	16.97	44.83	31.92
587440	597064	Putative Beta-1,3-glucosyltransferase	ME; EA	scaffold00944	47.55	32.82	26.54
596562	601020	Noncoding RNA	EA	scaffold00944	47.55	25.30	24.87
597069	598912	Chymotrypsin-2-like	EA	scaffold00944	47.39	25.30	24.87
606651	608619	Noncoding RNA	E+	scaffold00944	33.21	14.06	21.01
866318	872452	Uncharacterized, similar to integumentary mucin C.1 protein (94% coverage, 99% identity, *D.* *magna*)	E+	scaffold00944	47.30	37.07	59.83
868009	868917	Uncharacterized	E+	scaffold00944	47.30	37.07	59.83
872613	877571	Uncharacterized	E+	scaffold00944	47.30	37.07	59.83
913115	920541	Noncoding RNA	E+	scaffold00944	33.87	19.77	45.27
915177	937472	Putative neuropeptide receptor	E+	scaffold00944	37.66	19.77	45.27
962327	980655	Rap1 GTPase-activating protein	E+	scaffold00944	37.66	17.91	11.74
1198279	1199273	Uncharacterized, similar to protein FAM98B-like (100% coverage, 96% identity, *D.* *magna*)	E+	scaffold00944	34.00	0.79	33.58
1199954	1206543	Disintegrin and metalloproteinase domain-containing protein 28	EA	scaffold00944	11.90	33.38	8.88
1274156	1284959	Anion exchange protein/Sodium bicarbonate transporter-like protein 11	ME; EA; E+	Resistance region	151.92	63.13	107.69
1308110	1311274	Uncharacterized	E+	Resistance region	32.30	23.83	48.18
1311330	1312503	Uncharacterized	E+	Resistance region	32.30	23.83	48.18
1331506	1334662	Hypothetical, homology with matrix metalloproteinase 1 (70% coverage, 59% identity, *Daphnia pulex*) and Galactose-3-O-sulfotransferase 2 (70% coverage, 43% identity *D.* *magna*)	ME; E+	Resistance region	32.30	23.83	48.18
1359580	1364008	Uncharacterized, possible homology with matrix metalloproteinase 1 (68% coverage, 58% identity, *Daphnia pulex*)	East	Resistance region	51.48	55.54	34.60
1370390	1372988	Putative metal-responsive transcription factor 1 protein	ME	Resistance QTL	43.42	18.51	10.02
1431210	1433374	Phytanoyl-CoA dioxygenase	ME; EA; E+	Resistance QTL	27.57	28.52	15.70
1494522	1497956	Beta-1,3-*N*-acetylglucosaminyltransferase	ME	Resistance QTL	23.81	59.30	69.64
1501990	1503649	Uncharacterized, similar to *N*-acetylneuraminate 9-O-acetyltransferase-like (79% coverage, 98.6% identity, *D.* *magna*)	E+	Resistance QTL	23.81	59.30	69.64
1503794	1504979	Alpha1,3 fucosyltransferase	E+	Resistance QTL	23.81	59.30	69.64
1505080	1510969	Putative WSC domain-containing protein 1 (sulfotransferase activity)	E+	Resistance QTL	23.81	59.30	69.64
1518381	1524265	Putative vascular endothelial growth factor receptor 3/brain chitinase and chia	E+	Resistance region	33.53	17.63	69.44
1639127	1641901	Uncharacterized	EA	scaffold00944	60.12	37.24	39.95
1639490	1640158	Noncoding RNA	EA	scaffold00944	60.12	37.24	39.95
1652260	1690450	Uncharacterized	EA	scaffold00944	73.78	43.65	50.26
1678117	1683180	Uncharacterized, similar to trypsin-like isoform X1 (*D.* *magna*), 100% coverage, 88.7% identity	EA	scaffold00944	73.78	43.65	50.26
1823839	1829476	Popeye domain-containing protein 3	ME	scaffold00944	32.02	10.68	19.79
1854814	1864203	Multidrug resistance-associated protein 7-like	EA	scaffold00944	13.29	27.78	12.63
1867949	1870932	Histone deacetylase 8	E+	scaffold00944	6.99	23.46	22.54
1885017	1888137	Clip-domain serine protease, similar to trypsin Blo t 3-like (100% coverage, 96.8% identity, *D.* *magna*)	E+	scaffold00944	15.86	30.42	36.06
1888238	1902202	High choriolytic enzyme/putative Metalloendopeptidase	E+	scaffold00944	43.75	38.63	18.50
1902029	1906857	Clip-domain serine protease/putative Trypsin-7	E+	scaffold00944	62.49	89.36	33.86
1907645	1910718	Clip-domain serine protease/putative Trypsin-7	E+	scaffold00944	60.72	107.84	58.13
1910898	1913791	High choriolytic enzyme/putative metalloendopeptidase	E+	scaffold00944	53.68	86.37	31.78
1953036	1963323	Lactosylceramide/alpha-1,4-*N*-acetylglucosaminyltransferase	E+	scaffold00944	19.62	39.60	80.49
1963325	1965982	Lactosylceramide. Similar to *N*-acetylneuraminate 9-O-acetyltransferase (99% coverage, 72.7% identity, *D.* *magna*)	E+	scaffold00944	27.28	48.74	42.63
1966223	1972322	Putative vascular endothelial growth factor, brain chitinase, and chia	ME; E+	scaffold00944	29.91	14.44	18.13
1971615	1981027	Brain chitinase and chia, similar to vascular endothelial growth factor (63% coverage 93.1% identity, *D.* *magna*)	E+	scaffold00944	30.21	23.25	17.48
1996043	1999375	Putative GMP synthase	ME	scaffold00944	42.88	10.16	13.79
2069556	2075588	Putative eukaryotic translation initiation factor 4B	ME	scaffold00944	60.45	10.05	7.76

Note.—For some uncharacterized proteins, a protein–protein BLAST search was performed at https://blast.ncbi.nlm.nih.gov/Blast.cgi to identify possible homologs. In those cases, we report the percentage of coverage, identity, and the species in which the homolog was found. For each gene, we highlight whether it was found in the original resistance QTL (excluding the supergene), in the region around the resistance supergene (QTL ± 100 kb), or elsewhere on scaffold00944. For each candidate, we also indicate the maximum value for the *B_0,__MAF_* statistics composite likelihood ratio in each of the three geographic groups (see also fig. 3).

## Conclusion

The *D. magna–P. ramosa* system has become a model for the study of antagonistic coevolution in natural population (Mitchell et al. 2004; Decaestecker et al. 2007; Goren and Ben-Ami 2013; Auld et al. 2016). The discovery of a major resistance supergene in the host genome (Routtu and Ebert 2015; Bento et al. 2017) has allowed us to further explore the evolution of this region and to test whether it is under balancing selection, as the model of Red Queen coevolution would predict. Our study demonstrates that the region around this supergene diversity is indeed maintained through balancing selection. Furthermore, variation in this region is ancestral and older than variation in other regions of the genome. For the first time, our analysis links large-scale phenotypic diversity for parasite resistance with the underlying genomic region for a host–parasite system. As in other systems with balancing selection, such as mating types and incompatibility alleles (Joly and Schoen 2011; Roux et al. 2013), we found high phenotypic and genetic diversity combined with the absence of a large-scale geographic pattern. Textbook examples of balancing selection in genes related to immune function typically lack functional evidence of the interaction between host resistance genes and a specific coevolving parasite. Mechanistically, balancing selection at the *Pasteuria* resistance locus may be maintained by a matching allele resistance matrix that links host and parasite genotypes on a functional level (Luijckx et al. 2013; Metzger et al. 2016). The host locus underlaying this matching allele matrix is part of the supergene (Bento et al 2017), which is the center of the current study.

Earlier studies that provide evidence for balancing selection at disease loci in hosts typically only speculate about the coevolving parasite or assume a community of different parasites that vary in space and time (hence diffuse coevolution, see Ebert and Fields 2020). This study tested for balancing selection in a region of the host genome known to interact specifically with the widespread, virulent bacterial parasite *Pasteuria ramosa*, but not with other parasites (Routtu and Ebert 2015; Krebs et al. 2017; Keller et al. 2019). Specific coevolution—which we believe explains the polymorphisms at the resistance supergene region examined here—is the heart of the Red Queen hypothesis of antagonistic coevolution, as it was originally proposed by Clarke (although not under the name Red Queen) (Clarke 1976; Hamilton 1980) and taken up by others (Hamilton 1980; Frank 1991; Tellier and Brown 2007). The simplicity of this model, combined with the fascinating complexity produced by the intricate interactions between antagonists, have made it frequently cited for antagonistic coevolution and further, for phenomena linked to coevolution, such as the evolution of genetic recombination (Hamilton et al. 1990; Lively 2010). The *Daphnia–Pasteuria* system is among the few systems where Red Queen model’s assumption of specific coevolution is demonstrated and shown with strong evidence (Decaestecker et al. 2007; Duneau et al. 2011; Luijckx et al. 2011, 2013). Our finding of long-term balancing selection on the *Pasteuria* resistance locus here further reinforces this key prediction of the Red Queen model for specific coevolution, leaving a strong impact on the genome of the host.

## Materials and Methods

### Spatial Variability in Resistance Phenotypes

For each of the 125 *D. magna* clones, seven resistance phenotypes (resistotypes) were obtained using the attachment test (Duneau et al. 2011). Resistance (failure of the parasite to attach to the host cuticle) was coded as “R,” and susceptibility (attachment), as “S” (Andras and Ebert 2013; Luijckx et al. 2013, Bento et al. 2020). Resistotypes are defined by the R–S sequence for the seven tests each host clone underwent (at least three replicates per host–parasite combination). Pairwise phenotypic distance between individuals was coded as 0 when resistotypes were the same and 1 when they differed. Pairwise genetic differences between individuals were estimated from genomic data (see below) using the relatedness function in VCFTOOLS v0.1.12b (Danecek et al. 2011), which computes the Ajk statistics (Yang et al. 2010). This statistic should vary between 0 (for pairs of unrelated individuals) and 1 (for an individual with itself). Resistotype frequencies were calculated for 23 population samples (supplementary table S5, Supplementary Material online). Distance measures, including all phenotypes, were estimated as a Euclidean distance, using each resistotype as a distinct dimension. IBD was assessed by Mantel tests with the ecodist package in R (v3.6.3) (Dray and Dufour 2007). dbMEM were also used to assess the geographical variables influencing resistotypes composition at the different spatial scales in our study (Legendre et al. 2015). Resistotypes presence/absence or abundance data were first Hellinger transformed to avoid overweighting rare resistotypes and significant linear trends were removed, using the adespatial package in R.

### Whole-Genome Resequencing

Genomic DNA was extracted from the 125 *D. magna* and one *D. similis* clone (three times selfed) as in Fields et al. (2015) (see supplementary table S6, Supplementary Material online for details). Individuals were treated with antibiotics to evacuate their guts and reduce DNA from microbiota and food following the protocol of Dukić et al. (2016). DNA was extracted using an isopropanol precipitation protocol. Paired-end 125 cycle sequencing was performed by the Quantitative Genomics Facility service platform at the Department of Biosystem Science and Engineering (D-BSSE, ETH) in Basel, Switzerland, on an Illumina HiSeq 2000. Read quality was assessed with FastQCv0.11.5 (http://www.bioinformatics.babraham.ac.uk/projects/fastqc, last accessed August 9, 2021), and Trimmomatic (v0.32) (Bolger et al. 2014) was subsequently used to remove low quality bases, sequencing adapter contamination and systematic base calling errors. Sequences were aligned using BWA MEM (v0.7.15) on the *D. magna* genome assembly (v. 2.4) (Li et al. 2009). We improved the quality of this reference for the resistance region by replacing a part of the original scaffold944 (length = 2,137,955 bp) with a PacBio contig from the same Xinb3 reference (supplementary fig. S1, Supplementary Material online) as described in a previous study (Bento et al. 2017); however, only minor differences between the analyses using the old and the updated reference were observed. We converted coordinates for the PacBio contig into coordinates on scaffold00944 by carrying a BLAST (v2.6.0) search analysis between the two sequences (Altschul et al. 1997). BAM alignment files were filtered for quality, and PCR duplicates were removed using PICARD tools (v 2.0.1, http://broadinstitute.github.io/picard/, last accessed August 9, 2021). SNP calling was performed simultaneously on all samples using freebayes (v. 0.9.15-1). Freebayes is a haplotype caller, which automatically performs indels realignment and base quality recalibration. VCF files were then filtered using VCFTOOLS v0.1.12b (Danecek et al. 2011) to include SNPs with a minimum quality of twenty, a minimum genotype quality of 30, a minimum depth of coverage of 8X/genotype, and a mean maximum sequencing depth of 70×. We removed polymorphic indels and homopolymers before using the vcfallelicprimitive script in vcflib (https://github.com/vcflib, last accessed August 9, 2021) to convert the haploid calls into pointwise SNPs. Only SNPs that passed filters in at least 90% of samples were included in subsequent analyses (no more than 10% missing data). For simplicity, contigs and scaffolds shorter than 10 kb were excluded from analyses of the genomic background (see supplementary table S6, Supplementary Material online for quality statistics after filtering). Poor mapping could lead to an excess of false-positive polymorphisms and an excess of heterozygotes in the resistance region. We further examined six quality statistics for the whole genome and the resistance region: the mapping quality of the reference and alternate alleles, the proportion of reference and alternate alleles supported by properly paired-ends reads, the ratio between depth of coverage at heterozygous sites normalized by individual depth of coverage, and sequencing depth. No substantial differences were observed between the resistance region and the rest of the genome (supplementary fig. S5, Supplementary Material online).

### Structure and Descriptive Statistics

To characterize population structure, we used DAPC in the R package adegenet (v2.1.2) to perform a clustering analysis (Jombart et al. 2010) on a set of 8978 SNPs with no missing data; these were thinned every 1,000 bp to limit the effects of linkage and of variation in SNP density (supplementary fig. S4, Supplementary Material online). DAPC first decomposes the variance in the data set into principal components (PC), then performs a discriminant analysis on these PC to identify the most likely genetic clusters. We selected the clustering model with the highest support using BIC and retained 14 PC that explained about 21.4% of the total variance and two of the linear discriminants. These numbers were chosen through a cross-validation procedure that suggested perfect assignation to clusters with a 0% mean-square error (Jombart and Collins 2015). Tajima’s *D*, *F_ST_ and d_xy_* were calculated for nonoverlapping 1-kb windows using the R package PopGenome (v2.2.5) (Pfeifer et al. 2014), and *D. similis* was used as an outgroup for computing Fu and Li’s *F*, and *D*. This windows length was chosen to ensure independence between windows, based on the rapid decay in linkage disequilibrium over 1,000 bp in *Daphnia* genome (supplementary fig. S4, Supplementary Material online). Windows with less than 5 segregating sites were excluded.

### Demographic Model

We fitted a demographic model on SNP data using the likelihood algorithm implemented in fastsimcoal2.6 (Excoffier and Foll 2011). The model consisted of one ancestral population that split into three with gene flow, and allowed one population size change after each split to reflect the recent postglacial expansion in *D. magna* (Fields et al. 2018). The three populations corresponded to the three geographical clusters identified by the DAPC. To obtain accurate spectra and limit the impact of missing data, we used a subset of 48 clones with less than 5% missing data (supplementary table S7, Supplementary Material online), covering the whole species range as well as common resistotypes. Migration rates, along with current effective population sizes and time since divergence between populations, were estimated from the joint folded AFS with 30 independent runs, and included 2,458,902 SNPs with no missing data. We estimated the total number of callable sites with the coverage tool in BEDTOOLS v2.25.0 to exclude genomic intervals covered at less than 10× depth in each single individual (Quinlan and Hall 2010). Each run used 40 cycles of likelihood optimization, with 100,000 coalescent simulations per cycle. We present the results from the run with the highest likelihood. Time in years and effective population size were obtained by assuming a mutation rate of 8.96 × 10^−9^ substitution/generation (Ho et al. 2020) and ten generations (asexual and sexual) per year (Haag et al. 2009). The same procedure was applied to 100 bootstrapped frequency spectra to obtain confidence intervals for all parameters.

### Forward-in-Time Simulations

To understand how diversity statistics in 1*-*kb windows may be affected by demography, variable proportion of mutations under balancing selection, and equilibrium frequency, we performed simulations using the forward-in-time simulator SLiM 3 (Haller and Messer 2019) for 125 diploid individuals drawn from three populations following the demographic model inferred by fastsimcoal2.6. For consistency with previous analyses of genetic diversity in *Daphnia magna*, we assumed a mutation rate of 8.96 × 10^−9^/generation (sexual + asexual combined), and a recombination rate of 6.78 × 10^−8^/sexual generation (Dukić et al. 2016), equivalent to 6.78 × 10^−9^/generation (sexual + asexual combined). For scenarios with balancing selection, 0.01% or 0.1% of new mutations were under NFDS. At equilibrium frequency, the selective coefficient *s* was equal to 0 and varied, so that *s *=* *0.005 * (*f*_eq_ − *f*_obs_)/*f*_eq_, where *f*_eq_ is the equilibrium frequency, and *f*_obs_ the frequency of the allele at a given generation in a given population. This results in a dynamic where *s* approaches 0.005 as *f*_obs_ approaches 0, and –0.005 as *f*_obs_ approaches 2 * f_*e*__q_. To shorten run times, we scaled all parameters inferred by fastsimcoal2.6 by a factor of 100: migration, mutation and recombination rates were multiplied by 100, whereas effective population sizes, times in generation, and selection coefficients were divided by the same factor. This scaling maintains constant parameters that control mutation-selection-drift balance, such as *Nμ*, *Nm*, *Nr*, and *Ns*, with *μ* the mutation rate, *m* the migration rate, *r* the recombination rate, *s* the selection coefficient, and *N* is the effective population size. Simulations were run without any demographic event for 10,000 generations (after scaling) to ensure that mutation-selection-drift balance was achieved. We ran 1,000 simulations for each combination of parameters, producing a VCF file for each; summary statistics were computed with PopGenome.

### Test for Neutrality and Composite Likelihood Ratio Test for Balancing Selection

To test for deviation from neutrality, we generated 10 million coalescent simulations with fastsimcoal without scaling parameters and converted the outputs into VCF files to obtain statistics with PopGenome. Divergence and diversity statistics were summarized through a PCA using the prcomp function in R. We then predicted PCA scores for windows in the resistance region and for SLiM3 simulations. Envelopes containing 95% of points for each category were obtained using the locfit package in R (https://cran.r-project.org/web/packages/locfit/index.html). Deviation from neutrality was estimated for each 1-kb window in the genome by counting the proportion of simulations with a higher score on the first PC axis. The resulting *P* values were Bonferroni-corrected. We also calculated the *B_0,_*_*MAF*_ statistics (Cheng and Degiorgio 2020) for each of the three geographical groups identified by DAPC. The statistics does not require specifying a window’s size. Allele count data were extracted from the VCF file using VCFTOOLS.

### Ancestral Recombination Graphs and Alleles Age

We conducted coalescent analyses using ARGweaver (Rasmussen et al. 2014; Hubisz et al. 2020) on the same 48 clones with less than 5% missing data that we used in the demographic analyses (supplementary table S7, Supplementary Material online). ARGWeaver estimates local recombination rates and time since coalescence along the genome by reconstructing genealogies along the genome as well as changes in their branching due to recombination events ARG. To limit computation time, we focused on three scaffolds larger than 2 Mb (PacBio contig + end of scaffold00944, scaffold00512, and scaffold00024) that belonged to distinct linkage groups. We used the VCF file as an input, which makes ARGWeaver estimate the phase for each diploid genome. We used the same mutation and recombination rates as those used in demographic inference and simulations (see above). We also allowed changes in effective population size over time, using results from our fastsimcoal2.6 inference as a prior. We set the number of time points at which coalescence events could happen at 10 and set the maximum coalescence time at 5 million generations. Because we are mostly interested in ancient balancing selection, we also set the –delta parameter at 0.00001 so that coalescence events were less biased toward recent times than with the default value. The algorithm was run over 6,000 iterations and the MCMC chain sampled every 30 iterations. Observation of the likelihood values showed that convergence was achieved after 2,000 MCMC iterations, which were discarded as burn-in. We then extracted time since TMRCA for each nonrecombining block, the minimal time since coalescence for half of the samples, the recombination rate, and the total length of genealogies. To obtain statistics over 1-kb windows, we averaged estimates across nonrecombining blocks using the package regioneR (v1.20.0) (Gel et al. 2015).

### Refined Scans for Balancing Selection

For each SNP we computed *β* score (Siewert and Voight 2017), a statistic that identifies SNPs of allele clusters that segregate at similar frequencies, a pattern associated with long-term balancing selection. The length of the windows we examined around each given SNP was chosen using the formula provided in Siewert and Voight (2017). The distribution of haplotypes sizes is exponential with rate parameter *T* * *ρ*, with *T* being the time since balancing selection and *ρ* the recombination rate. Assuming *T* = 3 × 10^6^ generations (which is about ten times older than the average coalescence time retrieved by ARGWeaver) and *ρ* = 6.78 × 10^−9^/generation, 95% of haplotypes flanking a selected site should be shorter than 147 bp. We used a window size of 125 bp on each side of each focal SNP (for a total size of 250 bp, option -w 250), which, assuming 10 generations/year, should guarantee the detection of events that occurred in the last 300,000 years. Because alleles with equilibrium frequencies below 0.1 are more likely to be erased by drift, the statistic was not reported for SNPs at frequencies beneath this threshold (option -m 0.1). We performed analyses within each of the three clusters identified by DAPC to minimize confounding effects of population structure and regional adaptation and included all 125 clones. For some candidate regions, we extracted genealogies from the ARGWeaver output that overlapped with the SNP with the highest *β* score, sampling a random genealogy from the post burn-in MCMC iterations. We also sampled 5,000 random genealogies across the scaffolds, estimated pairwise phylogenetic distances between all pairs of samples using the R package ape, and performed Mantel test between these distance matrices and the matrix of geographical distances between samples. These analyses were conducted using the R packages ape (Paradis et al. 2004) and ecodist.

### Identification of Candidate Genes

We identified a set of strong candidates for balancing selection by first selecting SNPs and genomic regions in the top 1% for *β* scores (threshold estimated using all scaffolds larger than 10 kb). A mappability score was estimated using GenMap (Pockrandt et al. 2020) (v1.0.2), with a score of 1 indicating no repetitive sequence at a given position. We replaced regions from scaffold00944 covered by the improved PacBio scaffold. We filtered out regions that had overlapping, repetitive content, that is, sequences of at least 125 bp (125-mers, length of a single Illumina read) and scores <1. We allowed for up to four mismatches between repeated 125-mers. To further eliminate possible issues with copy-number variants that could artificially inflate diversity, we performed a one-tailed test for an excess of heterozygotes in all 125 individuals and removed regions where SNPs harbored *P* values lower than 1 × 10^−4^. Windows satisfying these conditions, and genes overlapping them, were extracted using BEDTOOLS (Quinlan and Hall 2010). We also extracted the highest *B_0,__MAF_* value at each candidate gene using BEDTOOLS.

## Supplementary Material

Supplementary data are available at *Molecular Biology and Evolution* online.

## Supplementary Material

msab217_Supplementary_Data

## References

[msab217-B1] Altschul SF , Madden TL, Schäffer AA, Zhang J, Zhang Z, Miller W, Lipman DJ. 1997. Gapped BLAST and PSI-BLAST: a new generation of protein database search programs. Nucleic Acids Res. 25(17):3389–253402. 9254694 10.1093/nar/25.17.3389 PMC146917

[msab217-B2] Ameline C , Bourgeois Y, Vögtli F, Savola E, Andras J, Engelstädter J, Ebert D. 2021. A two-locus system with strong epistasis underlies rapid parasite-mediated evolution of host resistance. Mol Biol Evol. 38(4):1512–1528. 33258959 10.1093/molbev/msaa311 PMC8042741

[msab217-B3] Andras JP , Ebert D. 2013. A novel approach to parasite population genetics: experimental infection reveals geographic differentiation, recombination and host-mediated population structure in *Pasteuria ramosa*, a bacterial parasite of *Daphnia*. Mol Ecol. 22(4):972–986. 23279064 10.1111/mec.12159

[msab217-B4] Andras JP , Fields PD, Pasquier LD, Fredericksen M, Ebert D. 2020. Genome-wide association analysis identifies a genetic basis of infectivity in a model bacterial pathogen. Mol Biol Evol. 37(12):3439–3452. 32658956 10.1093/molbev/msaa173 PMC7743900

[msab217-B5251119] Auld SKJR, , Tinkler SK, , Tinsley MC. 2016. Sex as a strategy against rapidly evolving parasites. Proc R Soc B. 283(1845):20162226. 10.1098/rspb.2016.2226 PMC5204169 28003455

[msab217-B5] ΘBento G , Fields PD, Duneau D, Ebert D. 2020. An alternative route of bacterial infection associated with a novel resistance locus in the *Daphnia–Pasteuria* host–parasite system. Heredity 125(4):173–183. 32561843 10.1038/s41437-020-0332-x PMC7490384

[msab217-B6] Bento G , Routtu J, Fields P, Bourgeois Y, Du Pasquier L, Ebert D. 2017. The genetic basis of resistance and matching-allele interactions of a host-parasite system: the *Daphnia magna*-*Pasteuria ramosa* model. PLoS Genet. 13(2):e1006596. 28222092 10.1371/journal.pgen.1006596 PMC5340410

[msab217-B7] Bergelson J , Kreitman M, Stahl EA, Tian D. 2001. Evolutionary dynamics of plant R-genes. Science 292(5525):2281–2285. 11423651 10.1126/science.1061337

[msab217-B8] Bitarello BD , De Filippo C, Teixeira JC, Schmidt JM, Kleinert P, Meyer D, Andres AM. 2018. Signatures of long-term balancing selection in human genomes. Genome Biol Evol. 10(3):939–955. 29608730 10.1093/gbe/evy054 PMC5952967

[msab217-B9] Bolger AM , Lohse M, Usadel B. 2014. Trimmomatic: a flexible trimmer for Illumina sequence data. Bioinformatics 30(15):2114–2120. 24695404 10.1093/bioinformatics/btu170 PMC4103590

[msab217-B10] Bolnick DI , Stutz WE. 2017. Frequency dependence limits divergent evolution by favouring rare immigrants over residents. Nature 546(7657):285–288. 28562593 10.1038/nature22351

[msab217-B11] Bourgeois Y , Roulin AC, Müller K, Ebert D. 2017. Parasitism drives host genome evolution: insights from the *Pasteuria ramosa*–*Daphnia magna* system. Evolution 71(4):1106–1113. 28230237 10.1111/evo.13209

[msab217-B12] Burri R. 2017. Interpreting differentiation landscapes in the light of long-term linked selection. Evol Lett. 1(3):118–131.

[msab217-B13] Charlesworth B. 2013. Background selection 20 years on. J Hered. 104(2):161–171. 23303522 10.1093/jhered/ess136

[msab217-B14] Charlesworth B , Nordborg M, Charlesworth D. 1997. The effects of local selection, balanced polymorphism and background selection on equilibrium patterns of genetic diversity in subdivided populations. Genet Res. 70(2):155–174. 9449192 10.1017/s0016672397002954

[msab217-B15] Charlesworth D. 2006. Balancing selection and its effects on sequences in nearby genome regions. PLoS Genet. 2(4):e64. 16683038 10.1371/journal.pgen.0020064 PMC1449905

[msab217-B16] Cheng X , Degiorgio M. 2020. Flexible mixture model approaches that accommodate footprint size variability for robust detection of balancing selection. Mol Biol Evol. 37(11):3267–3291. 32462188 10.1093/molbev/msaa134 PMC7820363

[msab217-B17] Clarke BC. 1976. Genetic aspects of host-parasite relationships. In: Taylor AER, Muller RM, editors. The ecological relationship of host-parasite relationships.Oxford: Blackwell. p. 87–104.

[msab217-B18] Cornetti L , Fields PD, Van Damme K, Ebert D. 2019. A fossil-calibrated phylogenomic analysis of *Daphnia* and the Daphniidae. Mol Phylogenet Evol. 137:250–262. 31125657 10.1016/j.ympev.2019.05.018

[msab217-B19] Cruickshank TE , Hahn MW. 2014. Reanalysis suggests that genomic islands of speciation are due to reduced diversity, not reduced gene flow. Mol Ecol. 23(13):3133–3157. 24845075 10.1111/mec.12796

[msab217-B20] Danecek P , Auton A, Abecasis G, Albers CA, Banks E, DePristo MA, Handsaker RE, Lunter G, Marth GT, Sherry ST, et al 2011. The variant call format and VCFtools. Bioinformatics 27(15):2156–2158. 21653522 10.1093/bioinformatics/btr330 PMC3137218

[msab217-B21] Decaestecker E , Gaba S, Raeymaekers J. A M, Stoks R, Van Kerckhoven L, Ebert D, De Meester L. 2007. Host-parasite “Red Queen” dynamics archived in pond sediment. Nature 450(7171):870–873. 18004303 10.1038/nature06291

[msab217-B22] Dray S , Dufour AB. 2007. The ade4 package: implementing the duality diagram for ecologists. J Stat Softw. 22:1–20.

[msab217-B23] Dukić M , Berner D, Roesti M, Haag CR, Ebert D. 2016. A high-density genetic map reveals variation in recombination rate across the genome of *Daphnia magna*. BMC Genet. 17(1):137 27737627 10.1186/s12863-016-0445-7 PMC5064971

[msab217-B24] Duneau D , Luijckx P, Ben-Ami F, Laforsch C, Ebert D. 2011. Resolving the infection process reveals striking differences in the contribution of environment, genetics and phylogeny to host-parasite interactions. BMC Biol. 9:11. 21342515 10.1186/1741-7007-9-11 PMC3052238

[msab217-B158119] Ebert D, , Duneau D, , Hall MD, , Luijckx P, , Andras JP, , Du Pasquier L, , Ben-Ami F. 2016. A Population Biology Perspective on the Stepwise Infection Process of the Bacterial Pathogen Pasteuria ramosa in Daphnia. Adv Parasitol. 91:265–310. 27015951 10.1016/bs.apar.2015.10.001

[msab217-B25] Ebert D , Fields PD. 2020. Host–parasite co-evolution and its genomic signature. Nat Rev Genet. 21(12):754–768. 32860017 10.1038/s41576-020-0269-1

[msab217-B26] Eizaguirre C , Lenz TL, Kalbe M, Milinski M. 2012. Rapid and adaptive evolution of MHC genes under parasite selection in experimental vertebrate populations. Nat Commun. 3:621. 22233631 10.1038/ncomms1632 PMC3272583

[msab217-B27] Excoffier L , Foll M. 2011. Fastsimcoal: a continuous-time coalescent simulator of genomic diversity under arbitrarily complex evolutionary scenarios. Bioinformatics 27(9):1332–1334. 21398675 10.1093/bioinformatics/btr124

[msab217-B28] Fields PD , Obbard DJ, McTaggart SJ, Galimov Y, Little TJ, Ebert D. 2018. Mitogenome phylogeographic analysis of a planktonic crustacean. Mol Phylogenet Evol. 129:138–148. 29920335 10.1016/j.ympev.2018.06.028

[msab217-B29] Fields PD , Reisser C, Dukic M, Haag CR, Ebert D. 2015. Genes mirror geography in *Daphnia magna*. Mol Ecol. 24(17):4521–4536. 26190313 10.1111/mec.13324

[msab217-B30] Frank SA. 1991. Ecological and genetic models of host-pathogen coevolution. Heredity 67(Pt 1):73–83. 1917553 10.1038/hdy.1991.66

[msab217-B31] Fu YX , Li WH. 1993. Statistical tests of neutrality of mutations. Genetics 133(3):693–709. 8454210 10.1093/genetics/133.3.693 PMC1205353

[msab217-B32] Gel B , Díez-Villanueva A, Serra E, Buschbeck M, Peinado MA, Malinverni R. 2015. RegioneR: an R/Bioconductor package for the association analysis of genomic regions based on permutation tests. Bioinformatics 32:289–291. 26424858 10.1093/bioinformatics/btv562 PMC4708104

[msab217-B33] Gibson AK , Delph LF, Vergara D, Lively CM. 2018. Periodic, parasite-mediated selection for and against sex. Am Nat. 192(5):537–551. 30332578 10.1086/699829 PMC6812496

[msab217-B34] Goren L , Ben-Ami F. 2013. Ecological correlates between cladocerans and their endoparasites from permanent and rain pools: patterns in community composition and diversity. Hydrobiologia 701(1):13–23.

[msab217-B35] Haag CR , McTaggart SJ, Didier A, Little TJ, Charlesworth D. 2009. Nucleotide polymorphism and within-gene recombination in *Daphnia magna* and *D. pulex*, two cyclical parthenogens. Genetics 182(1):313–323. 19299338 10.1534/genetics.109.101147 PMC2674827

[msab217-B36] Haller BC , Messer PW. 2019. SLiM 3: forward genetic simulations beyond the Wright-Fisher model. Mol Biol Evol. 36(3):632–637. 30517680 10.1093/molbev/msy228 PMC6389312

[msab217-B37] Hamilton WD. 1980. Sex versus non-sex versus parasite. Oikos 35:282–290.

[msab217-B38] Hamilton WD , Axelrod R, Tanese R. 1990. Sexual reproduction as an adaptation to resist parasites (A review). Proc Natl Acad Sci U S A. 87(9):3566–3573. 2185476 10.1073/pnas.87.9.3566 PMC53943

[msab217-B39] Ho EKH , Macrae F, Latta LC, McIlroy P, Ebert D, Fields PD, Benner MJ, Schaack S. 2020. High and highly variable spontaneous mutation rates in *Daphnia*. Mol Biol Evol. 37(11):3258–3266. 32520985 10.1093/molbev/msaa142 PMC7820357

[msab217-B40] Hubisz MJ , Williams AL, Siepel A. 2020. Mapping gene flow between ancient hominins through demography-aware inference of the ancestral recombination graph. PLoS Genet. 16(8):e1008895. 32760067 10.1371/journal.pgen.1008895 PMC7410169

[msab217-B41] Joly S , Schoen DJ. 2011. Migration rates, frequency-dependent selection and the self-incompatibility locus in Leavenworthia (Brassicaceae). Evolution 65(8):2357–2369. 21790581 10.1111/j.1558-5646.2011.01300.x

[msab217-B42] Jombart T , Collins C. 2015. A tutorial for discriminant analysis of principal components (DAPC) using adegenet. R Vignette. Available from: https://adegenet.r-forge.r-project.org/files/tutorial-dapc.pdf. Accessed August 9, 2021.

[msab217-B43] Jombart T , Devillard S, Balloux F. 2010. Discriminant analysis of principal components: a new method for the analysis of genetically structured populations. BMC Genet. 11:94. 20950446 10.1186/1471-2156-11-94 PMC2973851

[msab217-B44] Jousimo J , Tack AJM, Ovaskainen O, Mononen T, Susi H, Tollenaere C, Laine AL. 2014. Ecological and evolutionary effects of fragmentation on infectious disease dynamics. Science 344(6189):1289–1293. 24926021 10.1126/science.1253621

[msab217-B45] Kaltz O , Shykoff JA. 1998. Local adaptation in host–parasite systems. Heredity 81(4):361–370.

[msab217-B46] Kaufman J. 2018. Unfinished business: evolution of the MHC and the adaptive immune system of jawed vertebrates. Annu Rev Immunol. 36(36):383–409. 29677478 10.1146/annurev-immunol-051116-052450

[msab217-B47] Keller D , Kirk D, Luijckx P. 2019. Four QTL underlie resistance to a microsporidian parasite that may drive genome evolution in its *Daphnia* host. bioRxiv.

[msab217-B48] Krebs M , Routtu J, Ebert D. 2017. QTL mapping of a natural genetic polymorphism for long-term parasite persistence in *Daphnia* populations. Parasitology 144(13):1686–1694. 28835307 10.1017/S0031182017001032

[msab217-B49] Laine AL , Burdon JJ, Dodds PN, Thrall PH. 2011. Spatial variation in disease resistance: from molecules to metapopulations. J Ecol. 99(1):96–112. 21243068 10.1111/j.1365-2745.2010.01738.x PMC3020101

[msab217-B50] Leffler EM , Gao Z, Pfeifer S, Ségurel L, Auton A, Venn O, Bowden R, Bontrop R, Wall JD, Sella G, et al 2013. Multiple instances of ancient balancing selection shared between humans and chimpanzees. Science 339(6127):1578–1582. 23413192 10.1126/science.1234070 PMC3612375

[msab217-B51] Legendre P , Fortin MJ, Borcard D. 2015. Should the mantel test be used in spatial analysis? Methods Ecol Evol. 6(11):1239–1247.

[msab217-B52] Li H , Handsaker B, Wysoker A, Fennell T, Ruan J, Homer N, Marth G, Abecasis G, Durbin R, 1000 Genome Project Data Processing Subgroup. 2009. The sequence alignment/map format and SAMtools. Bioinformatics 25(16):2078–2079. 19505943 10.1093/bioinformatics/btp352 PMC2723002

[msab217-B53] Lively CM. 2010. A review of Red Queen models for the persistence of obligate sexual reproduction. J Hered. 101(Suppl):S13–S20. 20421322 10.1093/jhered/esq010

[msab217-B54] Lively CM , Dybdahl MF. 2000. Parasite adaptation to locally common host genotypes. Nature 405(6787):679–681. 10864323 10.1038/35015069

[msab217-B55] Luijckx P , Ben-Ami F, Mouton L, Du Pasquier L, Ebert D. 2011. Cloning of the unculturable parasite *Pasteuria ramosa* and its *Daphnia* host reveals extreme genotype-genotype interactions. Ecol Lett. 14(2):125–131. 21091597 10.1111/j.1461-0248.2010.01561.x

[msab217-B56] Luijckx P , Fienberg H, Duneau D, Ebert D. 2012. Resistance to a bacterial parasite in the crustacean *Daphnia magna* shows Mendelian segregation with dominance. Heredity 108(5):547–551. 22167056 10.1038/hdy.2011.122 PMC3330695

[msab217-B57] Luijckx P , Fienberg H, Duneau D, Ebert D. 2013. A matching-allele model explains host resistance to parasites. Curr Biol. 23(12):1085–1088. 23707426 10.1016/j.cub.2013.04.064

[msab217-B58] Metzger CMJA , Luijckx P, Bento G, Mariadassou M, Ebert D. 2016. The Red Queen lives: epistasis between linked resistance loci. Evolution 70(2):480–487. 26763092 10.1111/evo.12854

[msab217-B59] Mitchell SE , Read AF, Little TJ. 2004. The effect of a pathogen epidemic on the genetic structure and reproductive strategy of the crustacean *Daphnia magna*. Ecol. Lett. 7(9):848–858.

[msab217-B60] Mouton L , Traunecker E, McElroy K, Du Pasquier L, Ebert D. 2009. Identification of a polymorphic collagen-like protein in the crustacean bacteria *Pasteuria ramosa*. Res Microbiol. 160(10):792–799. 19770039 10.1016/j.resmic.2009.08.016

[msab217-B61] Paradis E , Claude J, Strimmer K. 2004. APE: analyses of phylogenetics and evolution in R language. Bioinformatics 20(2):289–290. 14734327 10.1093/bioinformatics/btg412

[msab217-B62] Pfeifer B , Wittelsburger U, Ramos-Onsins SE, Lercher MJ. 2014. PopGenome: an efficient swiss army knife for population genomic analyses in R. Mol Biol Evol. 31(7):1929–1936. 24739305 10.1093/molbev/msu136 PMC4069620

[msab217-B63] Phillips KP , Cable J, Mohammed RS, Herdegen-Radwan M, Raubic J, Przesmycka KJ, van Oosterhout C, Radwan J. 2018. Immunogenetic novelty confers a selective advantage in host-pathogen coevolution. Proc Natl Acad Sci U S A. 115(7):1552–1557. 29339521 10.1073/pnas.1708597115 PMC5816137

[msab217-B64] Pockrandt C, Alzamel M, Iliopoulos CS, Reinert K. 220. GenMap: ultra-fast computation of genome mappability. Bioinformatics. 36(12):3687–3692. 10.1093/bioinformatics/btaa222 PMC7320602 32246826

[msab217-B65] Quinlan AR , Hall IM. 2010. BEDTools: a flexible suite of utilities for comparing genomic features. Bioinformatics 26(6):841–842. 20110278 10.1093/bioinformatics/btq033 PMC2832824

[msab217-B66] Rabajante JF , Tubay JM, Ito H, Uehara T, Kakishima S, Morita S, Yoshimura J, Ebert D. 2016. Host-parasite Red Queen dynamics with phase-locked rare genotypes. Sci Adv. 2(3):e1501548. 26973878 10.1126/sciadv.1501548 PMC4783124

[msab217-B67] Radwan J , Babik W, Kaufman J, Lenz TL, Winternitz J. 2020. Advances in the evolutionary understanding of MHC polymorphism. Trends Genet. 36(4):298–311. 32044115 10.1016/j.tig.2020.01.008

[msab217-B68] Rasmussen MD , Hubisz MJ, Gronau I, Siepel A. 2014. Genome-wide inference of ancestral recombination graphs. PLoS Genet. 10(5):e1004342. 24831947 10.1371/journal.pgen.1004342 PMC4022496

[msab217-B69] Rico Y , Morris-Pocock J, Zigouris J, Nocera JJ, Kyle CJ. 2015. Lack of spatial immunogenetic structure among wolverine (*Gulo gulo*) populations suggestive of broad scale balancing selection. PLoS One. 10(10):e0140170. 26448462 10.1371/journal.pone.0140170 PMC4598017

[msab217-B70] Routtu J , Ebert D. 2015. Genetic architecture of resistance in *Daphnia* hosts against two species of host-specific parasites. Heredity 114(2):241–248. 25335558 10.1038/hdy.2014.97 PMC4815634

[msab217-B71] Roux C , Pauwels M, Ruggiero M-V, Charlesworth D, Castric V, Vekemans X. 2013. Recent and ancient signature of balancing selection around the S-locus in *Arabidopsis halleri* and *A. lyrata*. Mol Biol Evol. 30(2):435–447. 23104079 10.1093/molbev/mss246 PMC3548311

[msab217-B72] Sackton TB , Lazzaro BP, Schlenke T. A, Evans JD, Hultmark D, Clark AG. 2007. Dynamic evolution of the innate immune system in Drosophila. Nat Genet. 39(12):1461–1468. 17987029 10.1038/ng.2007.60

[msab217-B74] Seefeldt L , Ebert D. 2019. Temperature- versus precipitation-limitation shape local temperature tolerance in a Holarctic freshwater crustacean. Proc Biol Sci. 286(1907):20190929. 31337313 10.1098/rspb.2019.0929 PMC6661336

[msab217-B75] Siewert KM , Voight BF. 2017. Detecting long-term balancing selection using allele frequency correlation. Mol Biol Evol. 34(11):2996–3005. 28981714 10.1093/molbev/msx209 PMC5850717

[msab217-B76] Tellier A , Brown JKM. 2007. Polymorphism in multilocus host-parasite coevolutionary interactions. Genetics 177(3):1777–1790. 17947440 10.1534/genetics.107.074393 PMC2147965

[msab217-B77] Tellier A , Brown JKM. 2011. Spatial heterogeneity, frequency-dependent selection and polymorphism in host-parasite interactions. BMC Evol Biol. 11(319):319. 22044632 10.1186/1471-2148-11-319 PMC3273489

[msab217-B78] Thrall PH , Barrett LG, Dodds PN, Burdon JJ. 2015. Epidemiological and evolutionary outcomes in gene-for-gene and matching allele models. Front Plant Sci. 6:1084. 26779200 10.3389/fpls.2015.01084 PMC4703789

[msab217-B79] Thrall PH , Laine AL, Ravensdale M, Nemri A, Dodds PN, Barrett LG, Burdon JJ. 2012. Rapid genetic change underpins antagonistic coevolution in a natural host-pathogen metapopulation. Ecol Lett. 15(5):425–435. 22372578 10.1111/j.1461-0248.2012.01749.x PMC3319837

[msab217-B80] Yampolsky LY , Zeng E, Lopez J, Williams PJ, Dick KB, Colbourne JK, Pfrender ME. 2014. Functional genomics of acclimation and adaptation in response to thermal stress in *Daphnia*. BMC Genomics. 15:859. 25282344 10.1186/1471-2164-15-859 PMC4201682

[msab217-B81] Yang J , Benyamin B, McEvoy BP, Gordon S, Henders AK, Nyholt DR, Madden PA, Heath AC, Martin NG, Montgomery GW, et al 2010. Common SNPs explain a large proportion of the heritability for human height. Nat Genet. 42(7):565–569. 20562875 10.1038/ng.608 PMC3232052

