# Supplementary material for: Balancing Selection for Pathogen Resistance Reveals an Intercontinental Signature of Red Queen Coevolution
Source: Mol Biol Evol. 2021 Jul 21;38(11):4918–33. doi: 10.1093/molbev/msab217 (PMC8557431; doi:10.1093/molbev/msab217)
Supplement: msab217_Supplementary_Data [file msab217_supplementary_data.zip › Supp_Methods_MBE_version.docx]

SUPPLEMENTARY TABLES

Table 1. Origin of *Pasteuria ramosa* strains used in this study.

| **Strain** | **Geographic region** | **Origin in the field** | **Cloned in host clone** | **Sampling date** | **Type** |
| --- | --- | --- | --- | --- | --- |
| C1 | Russia, Moscow | Single female infected from pond sediment | HO2 | 1996 | Clone |
| C19 | Germany, Gaarzerfeld | One infected female | Xinb3 | 1997 | Clone |
| P15 | Belgium | Passaged from a mixture of 8 lines from 8 females | Xinb3 | 2003 | Isolate |
| P20 | Switzerland | Infected female | - | 2012 | Isolate |
| P21 | Switzerland | Infected female | - | 2012 | Isolate |

Table 2. Mantel tests results for association between pairwise distance of individual resistotypes, genetic relatedness and geographic distance. *P*-values are obtained from 1,000,000 permutations and are not corrected for multiple testing.

| **Trait** | **Mantel *R*** | ***P*-value (left-sided)** | ***P*-value (right-sided)** | ***P*-value (two-sided)** |
| --- | --- | --- | --- | --- |
| Genetic relatedness | -0.56 | 1 | <10^-6^ | <10^-6^ |
| C1 resistotype | -0.04 | 0.92 | 0.08 | 0.19 |
| C19 resistotype | -0.03 | 0.75 | 0.25 | 0.48 |
| P15 resistotype (throat) | 0 | 0.45 | 0.54 | 0.98 |
| P15 resistotype (hindgut) | 0.019 | 0.22 | 0.78 | 0.48 |
| P20 resistotype | -0.05 | 0.989 | 0.0115 | 0.07 |
| P21 resistotype (throat) | 0.01 | 0.39 | 0.61 | 0.91 |
| P21 resistotype (hindgut) | 0.05 | 0.1 | 0.9 | 0.16 |
| All combined resistotypes | -0.01 | 0.66 | 0.34 | 0.67 |

Table 3. Mantel tests results for association between resistotypes frequencies, genetic relatedness and geographic distance for 23 populations with at least 5 phenotyped individuals. *P*-values are obtained from 1,000,000 permutations and are not corrected for multiple testing.

| **Trait** | **Mantel R** | **P-value (left-sided)** | **P-value (right-sided)** | **P-value (two-sided)** |
| --- | --- | --- | --- | --- |
| Genetic relatedness | -0.65 | 1 | <10^-6^ | <10^-6^ |
| C1 resistotype | 0.013 | 0.39 | 0.61 | 0.90 |
| C19 resistotype | 0.2 | 0.07 | 0.93 | 0.07 |
| P15 resistotype (throat) | 0.014 | 0.41 | 0.59 | 0.98 |
| P15 resistotype (hindgut) | -0.03 | 0.55 | 0.44 | 0.79 |
| P20 resistotype | 0.02 | 0.35 | 0.65 | 0.82 |
| All combined resistotypes | 0.053 | 0.27 | 0.73 | 0.62 |

Table 4. Point estimates and 95% confidence intervals for the Isolation-with-migration model inferred by fastsimcoal2.6. Parameters starting by “N” stand for effective population sizes (in number of diploid individuals). Parameters starting by “T” stand for times in years, assuming ten generations/year, and parameters starting by “mig” stand for migration rates. Population sizes with the “current” suffix designates modern parameters, while “anc” designates ancestral population sizes of the descendant populations designated by E+ (Europe+), ME and EA. Each of these three modern genetic clusters was allowed one change in effective population size at T_change years in the past. T_split parameters designate the time since the split between Europe+ and ME clusters, or between the ancestral population of these clusters and the East Asia cluster (T_split_E+_EA). The direction of the arrow in the migration parameters indicate the direction of gene flow forward in time.

| **Parameter** | **N_current_E+** | **N_current_EA** | **N_current_ME** | **Nanc_E+** | **Nanc_EA** | **Nanc_ME** | **Nanc_E+_ME** | **Nanc_E+_ME_EA** |  |  |  |
| --- | --- | --- | --- | --- | --- | --- | --- | --- | --- | --- | --- |
| Point estimate | 277633 | 261358 | 56214 | 132753 | 77122 | 96376 | 48684 | 38599 |  |  |  |
| Lower 2.5% | 259803 | 212546 | 51922 | 56231 | 63619 | 17833 | 32360 | 31242 |  |  |  |
| Upper 97.5% | 306863 | 270900 | 60089 | 202180 | 93079 | 198262 | 70732 | 45212 |  |  |  |
| **Parameter** | **T_change_E+** | **T_change_EA** | **T_change_ME** | **T_split_E+_ME** | **T_split_E+_EA** | **mig_EA->E+** | **mig_E+->EA** | **mig_ME->E+** | **mig_E+->ME** | **mig_ME->EA** | **mig_EA->ME** |
| Point estimate | 4116.7 | 3493.3 | 7181.4 | 8858.8 | 14014.7 | 4.99E-07 | 1.77E-06 | 2.71E-06 | 1.20E-05 | 2.44E-07 | 1.93E-07 |
| Lower 2.5% | 2075.3 | 2868.8 | 3789 | 7168.8 | 13098.1 | 4.63E-07 | 1.63E-06 | 2.12E-06 | 1.02E-05 | 2.72E-08 | 1.03E-07 |
| Upper 97.5% | 6803.4 | 4965.4 | 8848.3 | 11860.7 | 15701.4 | 6.77E-07 | 2.27E-06 | 3.15E-06 | 1.41E-05 | 3.24E-07 | 3.40E-07 |

Table 5. Frequencies of resistotypes in 52 *Daphnia magna* populations.

Table 6. Coordinates, frequency of missing genotypes and mean depth of coverage after filtering for scaffolds included in the analysis (see supplementary Excel file).

Table 7. List of clones included in ARGWeaver analyses (see supplementary Excel file).

Supplementary Figures

Figure 1. Population structure and history inferred from the 125 *D. magna* clones. A: Sites of origin and DAPC on 8,978 genome-wide SNPs with no missing data sampled every kb for 125 *D. magna* genotypes. The DAPC analysis identified three major groups: Europe+ (E+), East-Asia (EA) and Middle-East (ME). B: Best parameters inferred by Fastsimcoal2.6 for an Isolation-with-migration model of the three genetic groups. Migration rates correspond to the proportion of alleles in the receiving population that come from the source population.

Figure 2. BLAST alignment of PacBio contig and scaffold 944 (*Daphnia magna* genome, v 2.4)


Figure 3. Summary of SliM3 simulations for NFDS showing four summary statistics for each simulated population. Boxplots represent the distribution of values obtained for 1000 simulations under a given set of parameter values. Variable parameters include the equilibrium frequencies (f_eq_=0.1, 0.25 and 0.5) of polymorphisms under NFDS and the proportion of new mutations recruited by selection (frac=0.01 % and 0.1 %). The median values for each statistics within the resistance region are shown with horizontal lines. Values observed for the resistance region are also reported (second boxplot from the left).

Figure 4. Average LD-decay for biallelic SNPs separated by at most 1000bp for the whole genome (excluding the resistance region) and the resistance region (QTL+/-100kb). LD statistics were computed using the –r2 option in VCFTOOLS v0.1.12b (Danecek et al. 2011).

Sup Figure 5: Distributions of quality statistics for filtered SNPs called by freebayes. For the resistance region and the genomic background, we show the mapping quality of reference and alternate alleles (top row) and the support provided by paired-end reads for each allele (middle row). We also indicate the average ratio between sequencing depth at heterozygous genotypes and the individual sequencing depth (averaged over all genotypes). The total sequencing depth for all individuals is also indicated (bottom right panel).
